# Artificial Intelligence (AI) and Drug-Induced and Idiosyncratic Cytopenia: The Role of AI in Prevention, Prediction, and Patient Participation

**DOI:** 10.3390/hematolrep17030024

**Published:** 2025-04-29

**Authors:** Emmanuel Andrès, Amir El Hassani Hajjam, Frédéric Maloisel, Maria Belén Alonso-Ortiz, Manuel Méndez-Bailón, Thierry Lavigne, Xavier Jannot, Noel Lorenzo-Villalba

**Affiliations:** 1 Service de Médecine Interne, Hôpital de Hautepierre, Hôpitaux Universitaires de Strasbourg, 67000 Strasbourg, France; 2 Centre de Compétence des Cytopénies du Bas–Rhin, Hôpitaux Universitaires de Strasbourg, 67000 Strasbourg, France; 3 Laboratoire de Nanomédecine Imagerie et Thérapeutique, Université de Technologie de Belfort Montbéliard, 25200 Belfort-Montbéliard, France; 4 Service d’Hématologie, Clinique Saint-Anne, 67000 Strasbourg, France; 5 Servicio de Medicina Interna, Hospital Universitario de Gran Canaria Dr Negrin, 35010 Las Palmas de Gran Canaria, Spain; belalor@yahoo.es; 6 Servicio de Medicina Interna, Hospital Universitario Clínico San Carlos, 28040 Madrid, Spain; 7 Service d’Hygiène Hospitalière et Pôle de Santé Publique, Hôpital Civil, Hôpitaux Universitaires de Strasbourg, 67000 Strasbourg, France

**Keywords:** artificial intelligence, cytopenia, medication, idiosyncrasy, anemia, thombopenia, neutropenia, prevention, prediction, patient

## Abstract

Drug-induced and idiosyncratic cytopenias, including anemia, neutropenia, and thrombocytopenia, present significant challenges in fields like immunohematology and internal medicine. These conditions are often unpredictable, multifactorial, and can arise from a complex interplay of drug reactions, immune abnormalities, and other poorly understood mechanisms. In many cases, the precise triggers and underlying factors remain unclear, making diagnosis and management difficult. However, advancements in artificial intelligence (AI) are offering new opportunities to address these challenges. With its ability to process vast amounts of clinical, genomic, and pharmacovigilance data, AI can identify patterns and risk factors that may be missed by traditional methods. Machine learning algorithms can refine predictive models, enabling earlier detection and more accurate risk assessments. Additionally, AI’s role in enhancing patient engagement—through tailored monitoring and personalized treatment strategies—ensures more effective follow-up and improved clinical outcomes for patients at risk of these potentially life-threatening conditions. Through these innovations, AI is paving the way for a more proactive and personalized approach to managing drug-induced cytopenias.

## 1. Introduction

Drug-induced and idiosyncratic cytopenias—encompassing anemia, neutropenia, and thrombocytopenia—represent a significant and persistent challenge for healthcare professionals across all medical disciplines [1,2,3,4,5]. These hematologic disorders are often unpredictable and arise from a complex interplay of factors, including immune-mediated mechanisms, bone marrow suppression, direct toxicity, or hypersensitivity reactions. In many cases, the underlying pathophysiological mechanisms remain only partially understood, justifying the term “idiosyncratic” [4,5,6].

The spectrum of drugs implicated in the onset of these cytopenias is exceptionally broad and continues to expand, reflecting the diversity of therapeutic classes and clinical contexts involved. However, certain molecules are repeatedly and consistently associated with these adverse events, such as cotrimoxazole, carbimazole, clozapine, deferiprone, dapsone, ticlopidine, and noramidopyrine, among others [1,2,3,4,5]. These agents exemplify the unpredictability of drug-induced cytopenias, where onset can occur at any point during treatment—often without prior warning—and may lead to severe, life-threatening complications if not promptly recognized and managed.

In recent years, the integration of artificial intelligence (AI) into clinical research and medical decision making has opened promising perspectives for the early detection and prevention of these hematologic toxicities. Thanks to its capacity for large-scale data analysis, pattern recognition, and predictive modeling, AI-based systems offer new tools to identify at-risk patients, refine pharmacovigilance strategies, and enhance the personalization of treatment plans [7,8]. Beyond merely supporting clinicians, these technologies also hold the potential to empower patients by enabling real-time monitoring through connected devices, improving adherence to surveillance protocols, and reducing diagnostic delays.

Ultimately, the combination of robust clinical vigilance, technological innovation, and proactive patient education will be central to addressing the ongoing challenge posed by drug-induced cytopenias.

This manuscript provides a state-of-the-art review of AI and drug-induced and idiosyncratic cytopenias, with a focus on its role in prevention, prediction, and patient involvement.

## 2. Methods

The present work consists of a narrative review aimed at the state of the art regarding AI in the field of cytopenias, particularly drug-induced and idiosyncratic cytopenias.

An exhaustive search was carried out in the PubMed, Embase, and Cochrane Library databases up to March 2025. Keywords or phrases used included “artificial intelligence”, “cytopenia”, “anemia”, “thrombocytopenia”, “neutropenia”, “agranulocytosis”, “thrombotic microangiopathy”, “prevention”, “prediction”, and “participation”. The search was extended to commercial sites dedicated to artificial intelligence solutions for hematology, in particular cytopenias, using Google and Google Scholar.

Single case studies and unpublished works or sites not in English or French were excluded. Selected articles and sites were reviewed by two practitioners. Discrepancies were resolved by consensus or by consultation with a third researcher.

## 3. Discussion

### 3.1. Definition and Pathophysiology of Drug-Induced and Idiosyncratic Cytopenias

Idiosyncratic drug-induced cytopenias result from decreases in the number of blood cells such as red blood cells (anemia), neutrophils (neutropenia) or platelets (thrombocytopenia), or even several types of hematological cells (thrombotic microangiopathy), resulting from unpredictable reactions to drugs, unrelated to the dose administered or the known pharmacological properties of the drug. IDIA refers to a rare (6–10 cases per million of habitants), severe drop in neutrophil count caused by an adverse drug reaction in certain individuals, where the reaction occurs due to an individual’s unique genetic makeup, immune system, or other unknown factors [1,2]. The exact mechanisms are often poorly understood, but may include:

- Immunological reactions: The drug or its metabolites may modify cellular antigens, resulting in an immune response against blood cells;
- Direct toxicity: Some drugs can have a direct toxic effect on hematopoietic cells or bone marrow;
- Genetic predisposition: Individual genetic variations may make some people more susceptible to developing cytopenias in response to certain drugs.

Drug-induced neutropenia is a potentially serious and life-threatening adverse event that may occur secondary to therapy with a variety of agents. Cytotoxic chemotherapy can cause a predictable and dose-related decrease in neutrophil count.

### 3.2. Leveraging Genetic Data and AI to Understand and Predict Drug-Induced Cytopenias

To better understand the biological and molecular bases of drug-induced or idiosyncratic cytopenias, it is essential to explore the underlying genetic, immune, and cellular mechanisms that contribute to these conditions. Idiosyncratic reactions to medications, such as drug-induced agranulocytosis or drug-induced cytopenias, are thought to involve a complex interplay between genetic predispositions and immune system abnormalities [4]. These reactions often occur due to genetic variations that affect drug metabolism or immune response, making some individuals more susceptible to these adverse reactions. Genetic polymorphisms in drug-metabolizing enzymes (such as CYP450 enzymes) and immune-related genes (e.g., HLA alleles) have been identified as factors that predispose certain individuals to develop cytopenias when exposed to specific drugs [1,6].

At the molecular level, the pathogenesis of drug-induced cytopenias may involve immune-mediated mechanisms where the drug or its metabolites form complexes with cell surface proteins, triggering the immune system to attack hematopoietic cells (e.g., neutrophils, platelets) [6]. In some cases, drugs can induce the formation of drug-dependent antibodies or activate cytotoxic T cells that lead to the destruction of blood cells, resulting in cytopenias like neutropenia or thrombocytopenia.

With the increasing availability of large-scale genomic data collected from diverse genomic databases, pharmacogenomic studies, and electronic health records (EHRs), there is a growing opportunity to apply AI-driven approaches to predict, diagnose, and personalize treatment for patients at risk of drug-induced cytopenias [7]. By leveraging this vast amount of data, AI models can be trained to identify genetic risk factors, drug–gene interactions, and immune pathways that contribute to cytopenias. For instance, machine learning algorithms can analyze genomic data to detect genetic variants that predispose individuals to specific cytopenias in response to certain medications, helping clinicians to identify high-risk patients before prescribing these drugs. Additionally, integrating clinical data such as drug history, comorbid conditions, and lab results with genomic information can enhance the accuracy of AI models, making them more effective in predicting individual susceptibility to adverse drug reactions.

Incorporating this genomic data into AI systems can not only enhance the early detection of drug-induced cytopenias but also facilitate personalized medicine, where treatment decisions are made based on a patient’s genetic profile, significantly improving outcomes and minimizing the risk of adverse reactions [7]. Future studies and data-sharing initiatives that combine genetic databases, real-time clinical monitoring, and advanced AI algorithms will be crucial in defining new strategies to prevent and manage drug-induced cytopenias.

### 3.3. Enhancing Drug-Induced Cytopenia Prediction and Management Through AI and Machine Learning

To provide a clearer understanding of how AI learning and computing can be applied in the context of drug-induced cytopenias, this section will explore in more detail the specific machine learning (ML) techniques and computational frameworks utilized to analyze extensive genomic, clinical, and pharmacovigilance datasets. The following is a more comprehensive description of how AI can be leveraged in this area.

Machine learning algorithms have shown significant potential in predicting and analyzing adverse drug reactions (ADRs) like drug-induced agranulocytosis. These methods are able to process large and complex datasets from pharmacogenomics, clinical studies, and pharmacovigilance reports to identify novel risk factors, drug–drug interactions, and patient-specific vulnerabilities [8]. By analyzing genomic data, machine learning can uncover specific genetic markers that predispose individuals to adverse reactions, enabling more personalized approaches to treatment [9]. AI-driven models can integrate patient demographic information, clinical history, and genetic data to predict the likelihood of developing drug-induced cytopenias and facilitate early intervention [10].

Furthermore, AI techniques have been applied to large pharmacovigilance databases to improve signal detection for rare and idiosyncratic ADRs like agranulocytosis. By employing data mining and deep learning techniques, AI can identify subtle patterns in spontaneous ADR reports that may indicate emerging risks or drug combinations associated with agranulocytosis [11]. These technologies enable quicker recognition of potential safety signals, allowing for more timely regulatory actions and safer prescribing practices [12].

The integration of AI in drug safety is also crucial in creating predictive models for patients at risk of developing cytopenias. AI can be trained on historical patient data to build personalized risk profiles, taking into account genetic predispositions, current medications, age, sex, and comorbidities [13]. These models can be used to inform clinical decisions, allowing healthcare providers to adjust treatment plans based on individualized risk assessments [14]. As these models evolve and incorporate more diverse data sources, the potential for AI to transform the early detection and management of drug-induced cytopenias becomes more pronounced [15].

In summary, the application of AI and machine learning to drug-induced cytopenias is a rapidly evolving field that holds significant promise for improving drug safety. By harnessing the power of large-scale data analysis, AI can identify new risk factors, develop predictive models, enhance pharmacovigilance efforts, and ultimately enable more personalized and effective patient care [9,15].

### 3.4. Overview of AI in Cytopenias, Particularly Drug-Induced and Idiosyncratic Cytopenias

AI is a valuable tool in the management of cytopenias, particularly drug-induced and idiosyncratic forms [9]. In this field, AI offers opportunities for improved diagnosis, prediction, and personalized treatment.

AI can theoretically and potentially improve early detection of these conditions by analyzing laboratory test results and clinical data, often faster and more accurately than traditional methods [9,10,11,12,13,14,15,16,17,18]. Numerous academic and industrial studies are currently underway, with few results reported or published.

In hematology, the fields with the most accessible clinical data concern above all malignant pathologies such as lymphomas, myelomas, and other acute leukemias [19,20,21,22,23]. However, some clinical data are available in the field of myelodysplastic syndromes and bone marrow analysis using AI [22].

In the field of drug-induced and idiosyncratic cytopenias, AI can identify patterns and biomarkers associated with drug side effects, enabling better prediction of the risk of cytopenias occurring in patients treated with chemical agents (drugs) or biological agents (biotherapies, CART cells, etc.). In addition, predictive models fed by massive patient databases can estimate the probability of occurrence of these side effects and adjust treatments in real time.

AI analysis of a cohort of patients treated for drug-induced agranulocytosis at Strasbourg University Hospitals (n > 200) shows patterns at risk of unfavorable evolution: neutrophil count < 0.1 × 10^9^/L, elderly patients, renal failure, sepsis, septic shock (personal data).

For drug-induced and idiosyncratic cytopenias, where drug response varies between individuals, AI plays a key role in pharmacogenomics by analyzing patients’ genetic data to identify specific susceptibilities and avoid serious adverse reactions [6,7,8]. Until now, only two major histocompatibility complex loci, HLA-DQB1 (126Q) and HLA-B (158T), had been identified, using standard approaches, for clozapine neutropenia [4,5]. The risk of developing antithyroid-drug-induced agranulocytosis, particularly with carbimazole and methimazole, has been strongly linked to specific genetic predispositions involving human leukocyte antigen (HLA) alleles [1,2]. Notably, the presence of HLA-B*38:02 and HLA-DRB1*08:03 has been identified as a significant genetic risk factor in Asian populations, where some studies have reported odds ratios as high as 27 for carriers of these alleles undergoing treatment with antithyroid agents [3]. This strong association suggests an immuno-allergic mechanism, in which the drug or its metabolites may act as haptens, altering antigen presentation via the HLA complex and triggering an autoimmune response targeting neutrophils. These findings pave the way for a more personalized approach to therapy, where pretreatment genetic screening for high-risk HLA alleles could help identify susceptible individuals before drug exposure—much like the routine screening for HLA-B*57:01 to prevent abacavir hypersensitivity reactions. Integrating such genetic markers into clinical prescribing algorithms holds promise for reducing both the incidence and the severe outcomes of this potentially life-threatening adverse reaction [4,5]. In preliminary work, AI appears to identify many more.

The use of AI in this context goes beyond diagnosis, enabling dynamic patient monitoring, better personalization of treatments, and proactive risk management [16,17,18,19,20,21]. AI algorithms can also help predict complications associated with cytopenias, such as fever, serious infections (pneumonia, sepsis, gangrene, etc.), or bleeding (epistaxis, melaena, hematuria, menometrorrhagia, etc.), and suggest therapeutic adjustments to improve clinical outcomes.

Finally, these tools have the potential to accelerate the development of targeted therapeutic strategies (e.g., indications for hematopoietic growth factors) and to reinforce precision medicine in the management of drug-induced and idiosyncratic cytopenias [16,17,18,19,20,21].

### 3.5. AI for Personalized Prevention of Cytopenias, Particularly Drug-Induced and Idiosyncratic Cytopenias

The prevention of cytopenias, particularly drug-induced and idiosyncratic cytopenias, relies on a proactive approach aimed at identifying patients at risk before the onset of symptoms [1,2,3,4,5,6].

In this context, AI can play a key role by:

- Analyzing health data [16,17,18,19,20,21,22,23]: Based on electronic medical records (ontology), comorbidities, family history, and biological parameters, machine learning algorithms can detect early risk factors and alert clinicians;
- Predicting drug side effects [16,17,18,19,20,21,22,23]: Some cytopenias induced by drug treatments are expected, like those following chemotherapies, or totally unexpected, like drug-induced and idiosyncratic cytopenias (e.g., certain antibiotics like cotrimoxazole, synthetic antithyroid drugs like carbimazole, NSAIDs, clozapine, ticlopidine, etc.) [1,2,3,4,5,6].
- In this context, AI can help anticipate these reactions by analyzing patients’ genetic profiles and suggesting safer therapeutic alternatives;
- Personalizing prevention [16,17,18,19,20,21,22,23]: With the integration of diverse data streams—including genomic profiles, microbiome composition, lifestyle habits, and soon digitomics (continuous digital health data)—artificial intelligence (AI) is increasingly able to offer tailored medical recommendations. These can range from dietary adjustments and treatment optimization to personalized monitoring schedules. In particular, AI is a promising tool in interpreting complex pharmacogenomic data, including gene variants involved in drug metabolism (such as CYP450 enzymes or UGT1A1 polymorphisms). By leveraging machine learning, AI can help predict how an individual is likely to metabolize specific drugs, thus identifying both poor and ultra-rapid metabolizers before treatment initiation—a capability that holds potential to refine therapeutic decisions and prevent adverse drug reactions [24,25,26].

### 3.6. Finer Prediction Thanks to Machine Learning Models

AI plays a role in population medicine, analyzing large-scale data to identify trends and risks associated with cytopenias in groups of patients [16,17,18,19,20,21,22,23]. For example, by analyzing cohorts of patients treated for a particular pathology, AI can help identify common risk factors for drug-induced and idiosyncratic cytopenias [16,17,18,19,20,21,22,23].

This makes it possible to develop public health strategies aimed at preventing these conditions in specific populations, such as those at high risk or those taking specific treatments.

Drug-induced and idiosyncratic cytopenias are often difficult to anticipate due to their unpredictable nature. AI makes it possible to improve early detection and prediction of their occurrence thanks to:

- Analysis of blood biomarkers [16,17,18,19,20,21,22,23]: AI models can scan thousands of variables from biological analyses to identify precursor patterns of cytopenia before it becomes clinically apparent;
- Recognition of weak signals [16,17,18,19,20,21,22,23]: By processing longitudinal data, AI is able to identify small variations in blood counts that might be overlooked by the human eye but which herald an increased risk;
- Patient stratification [16,17,18,19,20,21,22,23]: Clustering algorithms can group patients into risk subgroups, enabling closer monitoring of the most vulnerable.

### 3.7. AI for Active Participation of Patients with Cytopenias, Particularly Drug-Induced and Idiosyncratic Cytopenias

Patient involvement is essential to improving the management of cytopenias, particularly drug-induced and idiosyncratic cytopenias. AI can promote this involvement through:

- Personalized monitoring applications [16,17,18,19,20,21,22,23,24]: Connected tools can monitor patients’ biological parameters in real time and inform them of any abnormalities. For example, a progressive drop in red blood cells could be signaled via an alert on a mobile application. Dematerialized questionnaires could be made available to patients to inform them of the presence of asthenia (anemia), hemorrhagic manifestations (thrombocytopenia), fever and/or infections (neutropenia), etc.;
- Education and autonomy [15,16,17,18,19,20,21,22]: Medical chatbots and conversational AI platforms enable patients to better understand their disease, obtain tailored advice, and quickly report unusual symptoms;
- Behavior and symptom analysis [16,17,18,19,20,21,22,23]: AI can cross-reference data from connected objects (smartwatches, scales, blood pressure monitors) with medical questionnaires to tailor recommendations and alert to early warning signs.

### 3.8. Other Areas of AI Development in Immuno-Hematology and Internal Medicine

AI has the potential to revolutionize hematology, immunology, and even internal medicine by improving the diagnosis, prognosis, and treatment of hemato- and/or immunological diseases [16,17,18,19,20,21]. However, it is important to stress that AI does not replace human expertise but complements it. The results obtained to date also need to be consolidated by prospective, larger-scale, real-life studies. Table 1 shows the main contributions of AI to these disciplines, highlighting the areas of application, the technologies used, and the specific contributions in each field (Table 1) [16,17,18,19,20,21,22,23].

A recent study using AI developed and validated a risk prediction model for moderate to severe bleeding in pediatric immune thrombocytopenia (ITP) [24]. Using prospective data from 286 children, the eXtreme Gradient Boosting (XGBoost) model outperformed other machine learning algorithms (AUC = 0.886). Key predictors identified via SHAP analysis included child age, age at diagnosis, and initial platelet count. The XGBoost model demonstrates strong predictive performance, potentially aiding in identifying high-risk pediatric ITP patients for improved clinical decision making. This novel model addresses the limitations of relying solely on platelet counts and adult-centric models. Xu et al. developed an interpretable machine learning algorithm that prospectively predicts the risk of thrombocytopenia in older critically ill patients in intensive care units (ICUs) that effectively predicts the risk (accuracy rate of 0.80 in the MIMIC database) and severity of postoperative thrombocytopenia [25]. Additionally, Nilius et al. created a friendly machine learning algorithm for the diagnosis of HIT (https://toradi-hit.org, access 26 February 2025) that was substantially more accurate than the currently recommended diagnostic algorithm and it could reduce delays in diagnosis and overtreatment in clinical practice [26].

Table 2 presents the main areas of development for cytopenias, detailing the potential contribution of AI to the management of anemias, thrombocytopenia, neutropenia, and other agranulocytoses, particularly drug-induced and idiosyncratic, again highlighting the areas of application, the technologies used, and the specific benefits in each context [16,17,18,19,20,21,22,23,24].

### 3.9. Potential Issues with Using AI in Hematology

The potential benefits of AI in hematology are undeniable, however, the inherent risks will have to be taken into consideration. Despite the impressive fidelity of synthetic data, the challenges of ensuring clinical accuracy and preserving the intrinsic relationships between data layers remain significant. While generative AI offers an innovative way to overcome the limitations of real-world data access, particularly for rare diseases, the complexity and variability of hematologic conditions present significant challenges for the widespread application of AI-generated data in clinical practice. Moreover, the ethical considerations of using these synthesized data, such as privacy concerns and the potential for bias, cannot be overlooked.

The reliance on AI for diagnostic and prognostic purposes brings concerns about model transparency, interpretability, and the potential for algorithms to perpetuate existing biases in healthcare data. AI enhances diagnostic accuracy, but there remains a need for human oversight to ensure that algorithms do not override clinical judgment. The risks of overreliance on AI, particularly in critical care settings, are real, and safeguards must be in place to mitigate the potential for errors or misdiagnosis. It would be timely and prudent to have a regulatory body oversee the development of AI models for machine learning, diagnostic algorithms, and patient privacy protection.

## 4. Conclusions: Towards an Integrated, Personalized Approach to Cytopenias

AI makes it possible to implement a more predictive, preventive, participatory, personalized, and population-based approach to the treatment of these disorders, contributing to more effective, patient-centered management [27]. At the same time, it brings the management of cytopenias into the realm of “P Medicine” [28].

Through its role in the analysis of clinical, genetic, and biological data, AI potentially enables better risk anticipation, optimized treatment management, and a reduction in complications associated with drug-induced and idiosyncratic cytopenias [16,17,18,19,20,21,22,23]. These theoretical data, or those based on preliminary studies or proofs of concept, need to be consolidated by clinical studies.

Thanks to its predictive analysis and care personalization capabilities, AI potentially represents a major advance in the management of cytopenias, particularly those that are drug-induced and idiosyncratic [16,17,18,19,20,21]. By combining targeted prevention, early detection, and active patient involvement, it should enable improved management of cytopenias and patients and optimized treatments.

However, for this technological revolution to be fully effective, it must be accompanied by rigorous clinical validation and seamless integration into medical practice. With this in mind, clinical studies are currently underway and are more necessary than ever.

AI does not replace the doctor’s expertise, but it does become a powerful ally in anticipating and better managing these complex pathologies [25].

## Figures and Tables

**Table 1:** Main contributions of artificial intelligence (AI) to Hematology, Immunology, and Internal Medicine [8,9,10,11,12,13,14,15].

Application	Technology Used	Specific AI Contributions
Early diagnosis of diseases	Computer vision, deep learning	Early detection of abnormalities in blood smears (leukemia, lymphoma), blood cell analysis.
Medical image analysis	AI for image analysis (MRI, CT, smears)	More accurate analysis of medical images, identification of subtle signs of hematological disease.
Predicting response to treatment	Predictive modeling, machine learning	Personalized treatment based on patients’ genetic and clinical characteristics.
Treatment optimization	Prediction algorithms, massive data analysis	Real-time monitoring of treatments, adjustment for side effects, improved clinical results.
Clinical data management	Big data, AI applied to case management	Management and organization of medical data, prevention of human error in prescriptions and treatments.
Research into new drugs	Machine learning, AI in biotechnology	Identification of new therapeutic compounds, identification of molecular targets for the treatment of blood diseases.
Monitoring disease progression	Predictive AI, time series analysis	Long-term patient monitoring, early detection of relapse, and treatment adjustment.
Analysis of genetic biomarkers	Supervised learning, AI for genomic analysis	Long-term patient monitoring, early detection of relapse, and treatment adjustment
Risk prediction and prevention	Statistical modeling, predictive analysis	Predicting the risk of hematological diseases in at-risk patients, personalized prevention.

**Table 2:** Main contributions of AI to cytopenias, with a focus on drug-induced and idiosyncratic cytopenias [16,17,18,19,20,21,22].

Pathology	Technology Used	Specific AI Contributions
Anemia Hb < 13.5 g/dL in men Hb < 12.0 g/dL in women	Supervised learning, clinical data analysis, AI for biomarker analysis	Faster, more accurate diagnosis of different types of anemia (iron deficiency, sickle cell anemia, megaloblastic anemia). Personalization of treatment according to underlying causes (iron deficiency, genetic disorders, etc.). Predict responses to treatment (e.g., responses to iron supplements or EPO).
Thrombopenia Platelets < 150 × 10^9^/L	Machine learning, AI for laboratory test analysis, predictive modeling	Early identification of drug-induced thrombocytopenia (induced by treatments such as heparin or chemotherapy). Real-time monitoring of thrombocytopenia in patients undergoing treatment. Predict the risk of serious complications (bleeding, thrombosis) linked to thrombocytopenia.
Drug-induced neutropenia - Neutrophils < 1.5 × 10^9^/L - Predictable and dose-related	Predictive modeling, AI for clinical and biological data analysis	Rapid detection of drug-induced neutropenia, particularly that induced by chemotherapy or antibiotics. Personalization of preventive treatment (e.g., antibiotic prophylaxis to avoid infections). Predict the risk of serious infections and immune reactions linked to neutropenia.
Idiosyncratic neutropenia - Neutrophils < 1.5 × 10^9^/L - Unpredictable, unrelated to the dose or pharmacological properties of the drug	Genetic data analysis, AI in pharmacogenomics	Identification of genetic susceptibility to idiosyncratic drug-specific neutropenia. Predict the risk of serious adverse reactions and personalize therapy to avoid them. Optimizing treatment through a better understanding of individual risk profiles and drug interactions.
